# Supplementary material for: Subjective Complaints and Coping Strategies of Individuals with Reported Low-Frequency Noise Perceptions
Source: J Clin Med. 2024 Feb 6;13(4):935. doi: 10.3390/jcm13040935 (PMC10889834; doi:10.3390/jcm13040935)
Supplement: Supplementary file 1 [file jcm-13-00935-s001.zip › jcm-2815390-supplementary.pdf]

Supplements

Table S1. Overall group differences between all groups on demographic variables

	X^2	*df*	*p*	*V*
Sex				
Females (%)	2.9	4	.58	.06
Education (%)	36.5	8	**<.001*** **	.14
Low	31.6	4	**<.001*** **	.19
Middle	6.1	4	.19	.08
High	4.6	4	.33	.07
Marital status (%)	37.1	24	**.043***	.21
Married[a]	12.0	4	**.02***	.12
Unmarried	12.1	4	**.02***	.12
No partner	7.6	4	.11	.09
Partner, living together	5.7	4	.22	.08
Partner, not living together	3.2	4	.53	.06
Divorced	.78	4	.94	.03
Widowed	13.0	4	**.01***	.12
	H	*df*	*p*	*η2*
Age in years	58.09	4	**< .001*** **	0.06

Note: * significant difference at a level p < .05. *** significant difference at a level p < .001.

Table S2. Single group differences on demographic variables

	LFN1 - LFN2-SA	*LFN1 - LFN2-SB*	*LFN1 - CG-SA*	*LFN1 - CG-SB*	*LFN2-SA - LFN2-SB*	*LFN-S2A - CG-SA*	*LFN2-SB - CG-SB*	*CG-SA - CG-SB*
Education: Low	**X2(1, N=312)**<br>**= 8.1,**<br>**p=.004****<br>**V=.16**	**X2(1, N=289)**<br>**= 8.8,**<br>**p=.003****<br>**V=.17**	**X2(1, N=410)**<br>**= 10.5,**<br>**p=.001****<br>**V=.16**	**X2(1, N=420)**<br>**= 13.4,**<br>**p= <.001*****<br>**V=.18**	X2(1, N=239)<br>= 0.8,<br>p= 0.36<br>V=.06	X2(1, N=360)<br>= 0.2,<br>p= 0.63<br>V=.03	X2(1, N=347)<br>= 0.9,<br>p= 0.34<br>V=.05	X2(1, N=468)<br>= 0.3,<br>p= 0.62<br>V=.02
Marital stat.: Married	**X2(1, N=312)**<br>**= 5.2,**<br>**p=.02***<br>**V=.13**	X2(1, N=289)<br>= 0.4,<br>p=.55<br>V=.04	X2(1, N=410)<br>= 1.5,<br>p=.22<br>V=.06	**X2(1, N=420)**<br>**= 5.4,**<br>**p= .02***<br>**V=.11**	**X2(1, N=239)**<br>**= 6.6,**<br>**p= .01***<br>**V=.17**	X2(1, N=360)<br>= 1.6,<br>p= 0.20<br>V=.07	**X2(1, N=347)**<br>**= 6.7,**<br>**p= 0.009****<br>**V=.14**	X2(1, N=468)<br>= 1.3,<br>p= 0.25<br>V=.05
Marital stat.: Unmarried	**X2(1, N=312)**<br>**= 4.9,**<br>**p=.03***<br>**V=.13**	X2(1, N=289)<br>= 0.26,<br>p=.61<br>V=.03	**X2(1, N=410)**<br>**= 6.8,**<br>**p=.009****<br>**V=.13**	**X2(1, N=420)**<br>**= 7.7,**<br>**p= .005****<br>**V=.14**	X2(1, N=239)<br>= 2.3,<br>p= 0.13<br>V=.10	X2(1, N=360)<br>= 0.0,<br>p= 0.99<br>V=.001	X2(1, N=347)<br>= 3.4,<br>p= 0.06<br>V=.10	X2(1, N=468)<br>= 0.02,<br>p= 0.88<br>V=.007
Marital stat.: Widowed	X2(1, N=312)<br>= 0.02,<br>p=.90<br>V=.007	**X2(1, N=289)**<br>**= 7.04,**<br>**p=.008****<br>**V=.16**	**X2(1, N=410)**<br>**= 7.0,**<br>**p=.008****<br>**V=.13**	X2(1, N=420)<br>= 2.8,<br>p=.09<br>V=.08	**X2(1, N=239)**<br>**= 6.2,**<br>**p= 0.01***<br>**V=.16**	**X2(1, N=360)**<br>**= 5.9,**<br>**p= 0.02***<br>**V=.13**	X2(1, N=347)<br>= 1.5,<br>p= 0.21<br>V=.07	X2(1, N=468)<br>= 1.3,<br>p= 0.26<br>V=.05
Age in years	U=10496,0<br>p=.08<br>r= -.10	U=9308,0<br>p=50<br>r= -.04	**U=14935,0**<br>**p= <.001*****<br>**r= -.24**	**U=16259,0**<br>**p= <.001*****<br>**r= -.21**	U=6497,5<br>p=.28<br>r= -.07	**U=9657.5**<br>**p= <.001*****<br>**r= -.30**	**U= 9677,0**<br>**p= <.001*****<br>**r= -.20**	U=26171,0<br>p=.41<br>r= -.04

Note: Group comparisons were only conducted between those groups that are being compared with each other within this article. Therefore, the group comparisons: LFN2-SA and CG-SB, and LFN2-SB and CG-SA were not conducted here. Significant results are highlighted in bold. * significant difference at a level p < .05. ** significant difference at a level p < .01. *** significant difference at a level p < .001.

Table S3. Proportion of missing values on all outcome variables for the three groups

	LFN1 N=181	LFN2-SA N=131	CG-SA N=229
	%	%	%
**Cognition**			
FLEI	0.6-6	0	0
BRIEF	4-7	0	0
**Depressive symptoms (BDI)**	2	0	0
		**LFN2-SB N=108**	**CG-SB N=239**
**Daily stress (APLN)**[a]			
All items	7-9	0	0 (4)[a]
Dependent items	3-6 (8)[a]	0 (2)[a]	0 (10)[a]
Independent items	4-5 (3)[a]	0 (2)[a]	0 (12)[a]
**Fatigue (FSS)**	0.6	0	0
**Sleep (PSQI)**			
Global	22	6	3
Subcomponents	0-11	0-5	0-3
**Coping**			
Active problem-oriented coping	1-7	0	0
Support Seeking	1-2	0	0
Avoidance Behavior	2-3	0	0
Non-dimension bound strategies	1-2	0	0
**Overall**	0-22	0-6	0-12
**Sum of categories with above average symptom reporting**	27	6[b]	3[b]

Note: [a] – The proportions in the brackets refer to those individuals, for whom no values could be provided for the APLN intensity scores. This was not due to missing data reporting, but since those individuals did not experience any stressors. [b] – refers to groups LFN2-SB and CG-SB, there were no missing values for groups LFN2-SA and CG-SA.

Table S4. Number and proportion of individuals scoring above the cut-offs of the BRIEF-A validity scales in the three groups

	**LFN1 N=181**	**LFN2-SB N=108**	**CG-SB N=239**
Negativity (%)	4 (2.2)	3 (2.8)	0 (0)
Infrequency (%)	1 (0.6)	13 (12.1)	25 (10.5)
Inconsistency (%)	3 (1.7)	1 (0.9)	1 (0.4)
**Overall (%)**	8 (4.4)	16 (14.8)	26 (10.9)

Table S5. Descriptives, significance tests and effect sizes on all outcome variables between the three groups excluding participants scoring above the BRIEF-A validity scale cut-offs

	**LFN1 n=181**				**LFN2 LFN2-SA=131**				**CG CG-SA=229**							LFN1 – LFN2	LFN1 – CG	LFN2 – CG
	N	M ± SD	Range	M	N	M ± SD	Range	M	N	M ± SD	Range	M	*H*	*p*	η^2	*r*	*r*	*r*
**Cognition**																		
FLEI Sum	161	42.9 ± 23.2	0-108	41	115	32.3 ± 18.1	2-81	31	203	20.5 ± 14.7	0-65	18	97.1	< .001**	.20	.25**	.52**	.32**
FLEI Attention	163	15.5 ± 8.7	0-36	15	115	11.0± 6.7	0-32	11	203	6.5 ± 5.3	0-24	5	109.9	< .001**	.23	.28**	.55**	.34**
FLEI Memory	169	15.6 ± 7.6	0-35	15	115	12.3 ± 6.1	1-28	12	203	8.4 ± 5.7	0-27	8	87.1	< .001**	.18	.23**	.49**	.31**
Executive functions																		
FLEI Executive functions	172	11.5 ± 7.6	0-37	10	115	9.0 ± 6.2	0-25	8	203	5.7 ± 4.7	0-20	4	66.7	< .001**	.13	.17*	.45**	.28**
BRIEF-A Global	159	108.8 ± 22.1	70-166	106	115	101.7 ± 20.8	71-162	100	203	91.4 ± 16.5	70-145	88	63.4	< .001**	.13	.22**	.45**	.22**
BRIEF-A BR	164	46.1 ± 9.8	30-77	45	115	43.5 ± 8.9	31-67	42	203	39.2 ± 7.6	30-67	38	53.2	< .001**	.11	.19**	.41**	.23**
BRIEF-A MC	164	62.4 ± 13.7	40-104	61	115	58.2 ± 13.2	40-95	57	203	52.1± 10.3	40-83	49	59.0	< .001**	.12	.22**	.44**	.20**
**Depressive symptoms**																		
BDI-II Sum	170	11.5 ± 8.2	0-42	10	115	7.5 ± 5.5	0-28	6	203	4.9 ± 4.3	0-27	4	82.9	< .001**	.17	.29**	.50**	.24**
					**LFN2-SB=108**				**CG-SB=239**									
**Daily stress (APLN)**																		
All items																		
Total	158	37.8 ± 32.4	0-172	26	108	41.7 ± 42.1	0-198	27	239	18.6 ± 35.7	0-248	8	108.2	< .001**	.21	.01	.48**	.40**
Frequency	161	26.3 ± 17.9	0-114	21	108	45.4 ± 31.5	1-114	37	239	22.2 ± 22.4	0-114	16	71.8	< .001**	.14	.32**	.20**	.43**
Intensity	157	1.4 ± 0.6	0.2-3.0	1.3	108	0.9 ± 0.5	0.0-2.3	0.8	229	0.7 ± 0.5	0.0-3.0	0.6	137.6	< .001**	.28	.44**	.58**	.21**
Dependent items																		
Total	164	8.3 ± 8.6	0-37	6	108	9.4 ± 10.6	0-50	5	239	4.1 ± 8.6	0-63	2	75.1	< .001**	.14	.01	.37**	.37**
Frequency	167	6.1 ± 5.1	0-28	5	108	10.9 ± 8.1	0-28	9	239	5.5 ± 5.7	0-28	4	53.3	< .001**	.10	.32**	.09	.39**
Intensity	150	1.3 ± 0.7	0.0-3.0	1.3	106	0.8 ± 0.5	0.0-2.0	0.7	215	0.6 ± 0.6	0.0-3.0	0.4	111.7	< .001**	.23	.42**	.53**	.21**
Independent items																		
Total	164	8.3 ± 6.4	0-37	7	108	8.5 ± 8.4	0-40	6	239	4.0 ± 7.5	0-52	2	106.1	< .001**	.21	.06	.48**	.37**
Frequency	166	5.3 ± 3.5	0-21	5	108	8.4 ± 5.9	0-21	6.5	239	4.3 ± 4.3	0-21	3	60.4	< .001**	.12	.26**	.22**	.39**
Intensity	159	1.6 ± 0.7	0-3.0	1.6	106	1.0 ± 0.6	0.0-2.7	1.0	211	0.7 ± 0.6	0.0-3.0	0.7	123.2	< .001**	.26	.44**	.55**	.18*

**Fatigue**																		
FSS Sum	173	36.7 ± 13.8	9-63	38	108	36.0 ± 10.7	13-62	37	239	27.1 ± 11.6	9-61	25	65.5	< .001**	.12	.05	.34**	.35**
**Sleep (PSQI)**																		
Global	137	8.6 ± 4.7	1-19	8	102	7.7 ± 4.0	0-19	7	231	5.5 ± 3.3	0-19	5	50.4	< .001**	.10	.08	.33**	.27**
Sleep Quality	170	1.6 ± 0.9	0-3	2	108	1.3 ± 0.8	0-3	1	239	0.9 ± 0.8	0-3	1	53.8	< .001**	.10	.17*	.36**	.19**
Sleep Latency	157	1.5 ± 1.1	0-3	1	103	1.5 ± 1.1	0-3	1	232	1.0 ± 1.0	0-3	1	29.6	< .001**	.06	.01	.25**	.22**
Sleep Duration	162	1.1 ± 1.1	0-3	1	106	0.9 ± 1.0	0-3	1	239	0.5 ± 0.8	0-3	0	31.2	< .001**	.06	.08	.27**	.19**
Habit Sleep Efficiency	161	1.5 ± 1.2	0-3	1	105	1.5 ± 1.3	0-3	1	236	1.1 ± 1.2	0-3	1	10.1	.006*	.02	.02	.15*	.11
Sleep Disturbance	155	1.5 ± 0.6	0-3	1	108	1.4 ± 0.5	0-3	1	239	1.2 ± 0.5	0-3	1	20.4	< .001**	.04	.06	.22**	.15*
Sleep Medication	173	0.8 ± 1.2	0-3	0	108	0.4 ± 0.9	0-3	0	239	0.3 ± 0.8	0-3	0	34.3	< .001**	.06	.19*	.28**	.08
Daytime Dysfunction	170	1.0 ± 0.9	0-3	1	108	0.8 ± 0.7	0-3	1	239	0.5 ± 0.6	0-3	0	39.3	< .001**	.07	.06	.29**	.23**

Note: LFN1 = Low frequency noise group recruited via LFN foundation, LFN2 = Low frequency noise group recruited via online panel, SA = subsample A filling out questionnaires regarding cognition and depressive symptoms, SB = subsample B filling out questionnaires regarding stress, coping, fatigue, and sleep, CG = Comparison group recruited via online panel, FLEI = Questionnaire for Complaints of Cognitive Disturbances, BRIEF-A = Behavior Rating Inventory of Executive Function – Adult Version, BRIEF-A BR = BRIEF-A Behavioral Regulation Index, BRIEF-A MC = BRIEF-A Metacognition, BDI = Beck Depression Inventory, APLN = Alledaagse Problemen Lijst, FSS = Fatigue Severity Scale, PSQI = Pittsburgh Sleep Quality Index, H = Kruskal-Wallis Statistic for testing overall group differences, $\eta2$ = eta squared. r – Effect size Cohen's r shown with the significance level derived from pairwise Mann Whitney U tests based on: * significant difference at a level $p < 0.01$, ** significant difference at a level $p < 0.001$, [light grey box] = medium effect size, [grey box] = large effect size.

Table S6. Proportions and number of individuals with above average symptom reporting, significance tests, and effect sizes on all outcome variables between the three groups excluding participants scoring above the BRIEF-A validity scale cut-offs

	**LFN1 n=181**		**LFN2 LFN2-SA=131**		**CG CG-SA=229**						LFN1 – LFN2	LFN1 – CG	LFN2 – CG
	N	%	N	%	N	%	$\chi 2$	*df*	*p*	*V*	*V*	*V*	*V*
**Cognition**[a]	**173**	**75.1**	**115**	**63.5**	**203**	**36.9**	**58.36**	**2**	**< .001****	**.35**	**.13**	**.38****	**.26****
FLEI Sum	161	61.5	115	46.1	203	20.2	65.75	2	< .001**	.37	.15	.42**	.27**
FLEI Attention	162	63.6	115	40.9	203	18.2	78.16	2	< .001**	.40	.23**	.46**	.25**
FLEI Memory	169	58.6	115	43.5	203	16.7	71.03	2	< .001**	.38	.15	.44**	.29**
Executive functions													
FLEI Executive functions	172	51.2	115	43.5	203	20.2	41.64	2	< .001**	.29	.08	.33**	.25**
BRIEF-A Global	159	23.9	115	14.8	203	5.4	25.66	2	< .001**	.23	.11	.27**	.16*
BRIEF-A BR	164	22.6	115	13.9	203	6.4	20.04	2	< .001**	.20	.11	.23**	.13
BRIEF-A MC	164	26.8	115	17.4	203	8.4	22.14	2	< .001**	.21	.11	.25**	.14
**Depressive symptoms (BDI-II)**	**170**	**17.1**	**115**	**4.3**	**203**	**1.0**	**37.00**	**2**	**< .001****	**.28**	**.19***	**.29****	**.11**
Moderate symptoms	170	12.9	115	4.3	203	1.0	24.35	2	< .001**	.22	.14	.24**	.11
Severe symptoms	170	4.1	115	0	203	0	13.29	2	.001*	.17	.13	.15*	-
			**LFN2-SB=108**		**CG-SB=239**								
**Daily stress (APLN)**	**159**	**61.0**	**108**	**77.8**	**239**	**29.7**	**80.40**	**2**	**< .001****	**.40**	**.18***	**.31****	**.45****
Total	158	40.5	108	41.7	239	11.7	54.57	2	< .001**	.33	.01	.33**	.34**
Frequency	161	45.3	108	75.0	239	28.9	64.47	2	< .001**	.36	.29**	.17**	.43**
Intensity	157	33.1	108	6.5	229	3.1	78.38	2	< .001**	.40	.32**	.41**	.08
**Fatigue (FSS)**	173	**55.5**	**108**	**58.3**	**239**	**26.4**	**48.44**	**2**	**< .001****	**.31**	**.03**	**.30****	**.31****
**Sleep (PSQI)**	137	**75.9**	**102**	**79.4**	**231**	**55.0**	**26.80**	**2**	**< .001****	**.24**	**.04**	**.21****	**.23****

Note: [a] Overall category in bold letters refers to participants who are categorized as reporting above average symptom reporting on at least one of the underlying variables. LFN1 = Low frequency noise group recruited via LFN foundation, LFN2 = Low frequency noise group recruited via online panel, SA = subsample A filling out questionnaires regarding cognition and depressive symptoms, SB = subsample B filling out questionnaires regarding stress, coping, fatigue, and sleep, CG = Comparison group recruited via online panel, FLEI = Questionnaire for Complaints of Cognitive Disturbances, BRIEF-A = Behavior Rating Inventory of Executive Function – Adult Version, BRIEF-A BR = BRIEF-A Behavioral Regulation Index, BRIEF-A MC = BRIEF-A Metacognition, BDI = Beck Depression Inventory, APLN = Alledaagse Problemen Lijst, FSS = Fatigue Severity Scale, PSQI = Pittsburgh Sleep Quality Index, % = Percentages from the total of valid cases, *V.* – Effect size Cramers V shown with the significance level of the group comparison based on: * significant difference at a level p < 0.01, ** significant difference at a level p < 0.001, [light grey] = medium effect size, [dark grey] = large effect size.

Table S7. Descriptives, significance tests and effect sizes on the Cope-Easy questionnaire between the three group excluding participants scoring above the BRIEF-A validity scale cut-offs

	LFN1 n=181				LFN2 LFN2-SB=108				CG CG-SB=239							LFN1 – LFN2	LFN1 – CG	LFN2 – CG
	N	M ± SD	Range	M	N	M ± SD	Range	M	N	M ± SD	Range	M	*H*	*p*	η^2	*r*	*r*	*r*
**Active problem-oriented coping[a]**	**162**	**24.6 ± 6.6**	**10-39**	**24**	**108**	**21.8 ± 7.2**	**10-40**	**21**	**239**	**21.0 ± 7.3**	**10-40**	**21**	**22.7**	**< .001****	**0.04**	**-0.20***	**-0.23****	**-0.04**
Active coping	171	5.9 ± 1.9	2-8	6	108	5.0 ± 1.9	2-8	5	239	4.8 ± 2.0	2-8	5	32.0	< .001**	0.06	-0.23**	-0.27**	-0.04
Suppression of competing activities	171	4.2 ± 1.9	2-8	4	108	4.0 ± 1.8	2-8	4	239	3.9 ± 1.8	2-8	4	2.1	.34	< 0.01	-0.04	-0.07	-0.03
Positive reframing	170	4.7 ± 2.0	2-8	4	108	4.3 ± 1.8	2-8	4	239	4.3 ± 1.9	2-8	4	4.9	.09	< 0.01	-0.09	-0.11	-0.02
Planning	170	5.8 ± 1.9	2-8	6	108	4.5 ± 1.8	2-8	4	239	4.5 ± 1.9	2-8	4	48.2	< .001**	0.09	-0.32**	-0.32**	<-0.01
Restrain	162	4.0 ± 1.6	2-8	4	108	4.0 ± 1.6	2-8	4	239	3.6 ± 1.6	2-8	3	9.0	.01	0.01	< 0.01	-0.14*	-0.12
**Support Seeking**	**170**	**14.9 ± 4.4**	**6-24**	**15**	**108**	**11.0 ± 4.0**	**6-22**	**10**	**239**	**10.0 ± 4.0**	**6-24**	**9**	**114.2**	**< .001****	**0.22**	**-.42****	**-0.51****	**-.14***
Instrumental support	171	4.6 ± 1.8	2-8	5	108	3.4 ± 1.6	2-8	3	239	3.0 ± 1.5	2-8	2	87.3	< .001**	0.17	-0.33**	-0.45**	-0.14
Focus on venting emotions	170	4.9 ± 1.8	2-8	5	108	3.7 ± 1.4	2-8	3	239	3.4 ± 1.5	2-8	3	79.5	< .001**	0.15	-0.35**	-0.42**	-0.11
Use of emotional support	170	5.4 ± 1.9	2-8	6	108	3.8 ± 1.6	2-8	4	239	3.5 ± 1.7	2-8	3	93.8	< .001**	0.18	-0.39**	-0.46**	-0.11
**Avoidance Behavior**	**169**	**10.8 ± 3.2**	**6-21**	**10**	**108**	**10.3 ± 3.3**	**6-23**	**10**	**239**	**9.1 ± 2.8**	**6-19**	**9**	**30.5**	**< .001****	**0.06**	**-.08**	**-0.27****	**-.17***
Self-distraction	170	4.7 ± 1.8	2-8	5	108	4.2 ± 1.8	2-8	4	239	3.6 ± 1.6	2-8	3	36.9	< .001**	0.07	-0.15	-0.30**	-0.14*
Behavioral disengagement	170	3.1 ± 1.5	2-8	2	108	3.0 ± 1.4	2-8	2	239	2.6 ± 1.0	2-8	2	9.6	.008*	0.02	-0.03	-0.15*	-0.11
Denial	170	3.0 ± 1.5	2-8	2	108	3.1 ± 1.6	2-8	2	239	2.8 ± 1.5	2-8	2	7.0	.03	0.01	-0.06	-0.09	-0.14
Non-dimension bound strategies																		
Religion	171	2.9 ± 1.7	2-8	2	108	3.0 ± 1.6	2-8	2	239	2.6 ± 1.5	2-8	2	7.8	.02	0.01	-.08	-.07	-.16*
Humor	169	3.9 ± 1.7	2-8	4	108	4.0 ± 1.7	2-8	4	239	3.7 ± 1.7	2-8	4	1.8	.41	< -0.01	-.04	-.04	-.07
Acceptance	170	4.7 ± 1.8	2-8	5	108	4.9 ± 1.6	2-8	5	239	4.8 ± 2.0	2-8	5	1.0	.61	< -0.01	-.06	<-.01	-.05
Substance use	170	4.9 ± 2.0	4-16	4	108	4.9 ± 1.5	4-11	4	239	4.3 ± .7	4-9	4	24.3	< .001**	.04	-.05	-.20**	-.24**

Note: [a] Scale scores are depicted in bold letters encompassing the underlying variables. LFN1 = Low frequency noise group recruited via LFN foundation, LFN2 = Low frequency noise group recruited via online panel, SB = subsample B filling out questionnaires regarding stress, coping, fatigue, and sleep, CG = Comparison group recruited via online panel, = Percentages from the total n of the groups, H = Kruskal-Wallis Statistic for testing overall group differences, η2 = eta squared. r – Effect size Cohen's r shown with the significance level derived from pairwise Mann Whitney U tests based on: * significant difference at a level p < 0.01, ** significant difference at a level p < 0.001, = medium effect size, = large effect size
